# Tailoring the Selective Permeation Properties of Asymmetric Cellulose Acetate/Silica Hybrid Membranes and Characterisation of Water Dynamics in Hydrated Membranes by Deuterium Nuclear Magnetic Resonance

**DOI:** 10.3390/membranes12060559

**Published:** 2022-05-28

**Authors:** Miguel P. da Silva, Maria J. Beira, Isabel D. Nogueira, Pedro J. Sebastião, João L. Figueirinhas, Maria Norberta de Pinho

**Affiliations:** 1 Center of Physics and Engineering of Advanced Materials (CeFEMA), Laboratory for Physics of Materials and Emerging Technologies (LaPMET), Instituto Superior Técnico (IST), Universidade de Lisboa (ULisboa), Av. Rovisco Pais 1, 1049-001 Lisboa, Portugal; miguel.pereira.da.silva@tecnico.ulisboa.pt (M.P.d.S.); maria.beira@tecnico.ulisboa.pt (M.J.B.); pedro.jose.sebastiao@tecnico.ulisboa.pt (P.J.S.); joao.figueirinhas@tecnico.ulisboa.pt (J.L.F.); 2 Department of Chemical Engineering (DEQ), Instituto Superior Técnico (IST), Universidade de Lisboa (ULisboa), Av. Rovisco Pais 1, 1049-001 Lisboa, Portugal; 3 Department of Physics (DF), Instituto Superior Técnico (IST), Universidade de Lisboa (ULisboa), Av. Rovisco Pais 1, 1049-001 Lisboa, Portugal; 4 MicroLab, Instituto Superior Técnico (IST), Universidade de Lisboa (ULisboa), Av. Rovisco Pais 1, 1049-001 Lisboa, Portugal; isabel.nogueira@tecnico.ulisboa.pt

**Keywords:** asymmetric CA/SiO_2_ membranes, phase inversion and sol-gel, water dynamics in confined media, deuterium relaxometry, ultrafiltration/nanofiltration

## Abstract

In this work, the water order and dynamics in hydrated films of flat asymmetric cellulose acetate (CA)/silica, CA/SiO_2_, and hybrid membranes, covering a wide range of nanofiltration (NF) and ultrafiltration (UF) permeation properties, were characterised by deuterium nuclear magnetic resonance (DNMR) relaxation. The range of NF/UF characteristics was attained by subjecting three CA/SiO_2_ membranes, prepared from casting solutions with different acetone/formamide ratios to drying post-treatments of solvent exchange and conditioning with surfactant mixtures. Post-treated and pristine CA/SiO_2_ membranes were characterised in terms of hydraulic permeability, selective permeation properties and molecular weight cut-off. These results were correlated with the DNMR relaxation findings. It was found that the post-treatment by solvent exchange caused membrane shrinkage that led to very different permeation characteristics and a significant enhancement of the DNMR relaxation observables. In contrast, conditioning with surfactant solutions exhibited a weaker effect over those properties. Scanning electron microscopy (SEM) images were obtained for the membranes post-treated with solvent exchange to confirm their asymmetric nature. This work provides an essential indication that DNMR relaxometry is a reliable tool to characterise the asymmetric porous structures of the NF/UF CA/SiO_2_ hybrid membranes.

## 1. Introduction

The development of the Loeb and Sourirajan [1] integrally skinned cellulose acetate (CA) membranes, prepared by the wet-phase inversion method, contributed to the widespread establishment of pressure-driven membrane processes of reverse osmosis (RO), nanofiltration (NF) and ultrafiltration (UF). Owing to the versatility of this preparation method, expressed in the formulation of the casting solutions combined with the control of casting conditions, membranes can be tailor-made across the RO-NF-UF range with diverse active and porous layer structures that determine their selective permeation properties [2,3,4]. This versatility is further enhanced in the new synthesis method of coupling the wet phase inversion technique with the sol-gel method to produce the novel monophasic hybrid asymmetric membranes of cellulose acetate (CA) and silica (SiO_2_), CA/SiO_2_ membranes, constituting the system of the present study [5,6]. Alongside the membranes’ active layer structure, the state of water within its porous matrix represents a crucial role in the elucidation of the mechanisms of membrane-selective transport. More so because the separation performance of a membrane is gauged by an interplay of contributing factors such as the pore size, electrical charge and hydrophilic/hydrophobic characteristics of the membrane polymeric or hybrid matrix and also of the solutes [7,8,9].

It is widely established that the structure and dynamical properties of water are substantially affected by the presence of confining solid surfaces [10,11]. Amongst the numerous cases of porous media where the state of water is crucial in their performance as far as selective mass transfer or catalytic reaction activity goes, the biomedical and pharmaceutical applications have special relevance. Savina et al. address this subject and report three states of water in cross-linked polymer and protein cryo-hydrogels [12]. In integrally skinned CA or cellulose esters membranes, the occurrence of water/polymer and water/water interactions has been investigated by microscopic and spectroscopic membrane characterisation to correlate between the structure of the membrane active layer, the surface morphology and the permeation characteristics [4,8,13,14]. These interactions are associated with specific water structures that rely on the hydrophobicity/hydrophilicity of the polymer’s solid surface, the time and conditions of exposure to water and the average pore diameter at the active layer membrane surface [4,15]. In particular, the pore size influences not only the extension to which the induced water dynamics and structure is propagated but also the type of interactions occurring at the water/polymer interface [16,17].

The pore geometry in porous media has been characterised by nuclear magnetic resonance (NMR) relaxation since the early work of S. D. Senturia and J. D. Robinson in the seventies of the last century [18]. When NMR relaxivities ($1/T_{1}$, $1/T_{2}$, $1/T_{1}\rho$) of a fluid confined in a porous system are significantly enhanced relative to the bulk fluid, the relaxivities enhancement is a good measure of the pore surface-to-volume ratio. Consequently, NMR has shown applicability as a probe of fluid flow permeability in porous systems [19]. The magnetic relaxation of deuterium (D) in deuterated water (D_2_O) is dominated by the interaction between the electric quadrupole moment of the nucleus and the local electric field gradients. The largest electric field gradient seen by the deuterium nucleus is that intrinsic to the D-O bond where it is located. Thus, relaxation is intramolecular in nature. The relaxation of D_2_O is enhanced in porous media; the rate of molecular rotation is reduced at solid surfaces, shifting it towards the Larmor frequency and thereby reducing the spin–lattice and spin–spin relaxation times, $T_{1}$ and $T_{2}$, respectively [20], which become reliable indicators of the water dynamics modification promoted by the confining surfaces.

The present work addresses the characterisation, by deuterium NMR (DNMR), of the water order and dynamics in hydrated films of CA/SiO_2_ membranes covering a wide range of UF and NF permeation properties, hydraulic permeability, molecular weight cut-off and preferential selectivity to salts and organic neutral macromolecules. To assure the large range of UF/NF, three UF CA/SiO_2_ membranes prepared from casting solutions with different acetone/formamide ratios were submitted to a drying process through post-preparation treatments of solvent exchange [21] and conditioning with surfactant mixtures [22]. Scanning electron microscopy (SEM) images were recorded for the solvent exchange post-treated membranes to verify their asymmetric nature. In this manner, the assessment of the effect of the drying post-treatments on the membranes structure modification and its selective permeation characteristics will be achieved.

## 2. Materials and Methods

### 2.1. Membrane Synthesis

A series of laboratory-made flat asymmetric CA/SiO_2_ hybrid membranes were synthesised by coupling the wet phase inversion technique [23] with the sol-gel method [24]. The methodology used is described by de Pinho et al. [5]. The membranes were prepared from casting solutions containing 16.4 wt.% of CA polymer, supplied by Sigma-Aldrich (Steinheim, Germany), varying ratios of formamide and acetone constituting the solvent system and a SiO_2_ content equal to 5 wt.%. During preparation, the acid catalysed hydrolysis of the SiO_2_ alkoxide sol-gel precursor was promoted in situ by adding deionised water, tetraethyl orthosilicate (TEOS), supplied by Sigma-Aldrich (Steinheim, Germany), and nitric acid to the polymer solution. All the chemicals were of reagent grade, and 65%-nitric acid was of technical grade. Thereafter, the solution was cast into a film with the aid of a 250 µm calibrated doctor blade and evaporated for 30 s before coagulation in ice-cold water. Table 1 shows the composition of the casting solutions and the film casting conditions used in the preparation of three different membranes with distinct UF porous structures, labelled as CA/SiO_2_-22, CA30/SiO_2_-30 and CA/SiO_2_-34. These membranes are identified throughout this text by a two-field code: the first field refers to the membrane hybrid matrix (CA/SiO_2_) followed by a second field code representing the numbers 22, 30 and 34 that correspond to the formamide contents of 21.3%, 29% and 32.9% (wt.%), respectively, in the casting solutions.

### 2.2. Membrane Drying Post-Treatments

To cover a wide range of UF and NF permeation characteristics, membranes cast from each formulation (CA/SiO_2_-22, CA/SiO_2_-30 and CA/SiO_2_-34 membranes) were subjected to a drying post-treatment by (i) solvent exchange or (ii) conditioning in surfactant mixtures. The treatment by (i) solvent exchange (SE) was carried out by using isopropanol/n-hexane combinations, as described by Lui et al. [21]. In that regard, the membrane samples were first immersed in aqueous solutions of successively higher alcohol contents (i.e., 25, 50, 75 and 100 vol.% isopropanol) for at least 24 h at each successive step. Afterwards, the membranes were immersed in isopropanol/n-hexane solutions of successively higher hexane contents (i.e., 25, 50, 75 and 100 vol.% n-hexane) for 24 h at each successive step. In the last step, the n-hexane was removed by evaporation in a desiccator at ambient temperature for 24 h. On the other hand, (ii) conditioning with surfactant mixtures was performed using solutions of non-ionic surface-active agents, triton X-100 and glycerol by adapting the procedure used by Vos et al. [22]. Succeeding membrane preparation, the films were immersed for 15 min in one of the following solutions: (a) an aqueous solution of glycerol 20 vol.% (G20), (b) in pure glycerol (G100) and (c) an aqueous solution of triton x-100 4 vol.% and glycerol 20 vol.% (GT). Subsequently, the membrane films were left hung up to dry, inside an *hotte*, under ambient conditions for 1 h. In the final stage, the membranes were placed inside a desiccator at ambient temperature for 24 h to ensure the evaporation of the reminiscent conditioning solution. Before being installed in the UF permeation cells, all CA/SiO_2_ post-treated membranes were washed in deionised water for 24 h to remove the remaining solvents from the treatments. Similarly, samples of untreated CA/SiO_2_ membranes from each formulation were kept immersed in deionised water for 24 h. All the chemicals used in the treatments were of reagent grade, and the conductivity of the deionised water was lower than 10 µS cm${}^{-1}$.

The identification of the dried post-treated membranes follows a three-field code: the first field code refers to the membrane hybrid matrix (CA/SiO_2_), the second is relative to the formamide content in the casting solutions (of 22, 30 and 34) and the third corresponds to the drying membrane post-treatment SE, G20, G100 and GT.

### 2.3. Membrane Permeation Experiments

**Figure 1:**
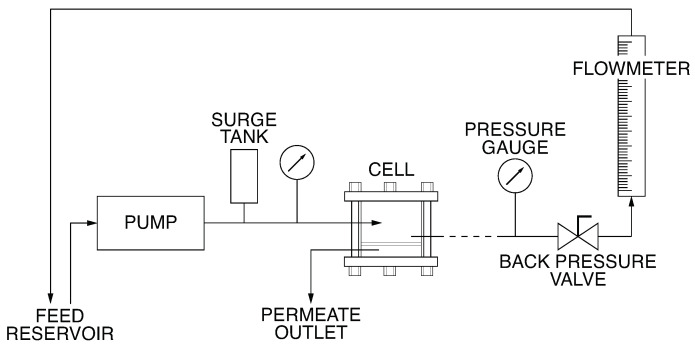
Flow diagram of the UF apparatus.

The characterisation of the permeation properties of the asymmetric CA/SiO_2_ hybrid membranes in terms of hydraulic permeability ($L_{P}$), apparent rejection coefficients to salts and neutral macromolecules (*f*), as well as the molecular weight cut-off (MWCO) was performed in a laboratory crossflow UF installation. Figure 1 shows the flow diagram of the UF apparatus used, which has been described previously [25].

A three-phase induction pump pressurises the system and imposes the feed flow rate. Succeeding, a surge tank reduces the pressure fluctuations in the cells assorted in series downstream of the pump. The operating gauge pressure, controlled by a back-pressure valve, is the average of the values measured by the inlet and outlet manometers that are placed before and after the series of permeation cells. Although only one cell is shown in Figure 1, five cells were used in the series so that five different membranes could be tested simultaneously. Each permeation cell has an effective membrane area of $13.2\times 10^{-4}$ m${}^{2}$, and its details are described by Sourirajan [26]. The pressure drop across each cell is below 0.08 bar.

Prior to the UF essays, all membranes were put through a pressure treatment, consisting of circulating distilled water on the high-pressure side of the membranes assembled in the UF cells at 4.2 bar, i.e., a pressure 20% higher than the highest operating pressure, for a period of 2–3 h. This treatment was intended to minimise compaction effects.

All ultrafiltration experiments were carried out at ambient laboratory temperature (20–30 °C).

The hydraulic permeability, $L_{P}$, corresponds to the slope of the linear variation of the pure water permeate flux, $J_{w}$, as a function of the applied transmembrane pressure, ΔP. The pure water permeates fluxes were measured at a feed flow rate of 2.5l min ${}^{-1}$, with transmembrane pressures ranging from 0.5 to 3.5 bar.

The data on all mass permeate fluxes were corrected to 25 °C using the relative viscosity and density of pure water.

Furthermore, the preferential selectivity of the membranes to salts and organic neutral macromolecules was computed in terms of an apparent rejection coefficient, *f*, defined as:

(1) $$f=\frac{\left[(solute\;concentration\;in\;the\;feed)-(solute\;concentration\;in\;the\;permeate)\right]}{\left(solute\;concentration\;in\;the\;feed\right)}$$

The permeation experiments were performed under total recirculation mode with a feed flow rate of 3.2 L min${}^{-1}$, a transmembrane pressure equal to 1 bar and a feed solute concentration of 600 ppm. In all the permeation experiments, the stabilisation time for each run was 30 min. The solutes used in these tests were salts, NaCl, MgCl_2_, Na_2_SO_4_ and MgSO_4_, and organic neutral macromolecules, polyethylene glycols (PEGs) of increasing molecular weights ranging from 200 to 35,000Da and dextran of 40,000Da. All the chemicals used were of reagent grade. The feed concentration was taken as the average of the values relative to feed samples collected at the beginning and at the end of each solute run. The concentration of salts was determined by conductivity measurements using a Crison GLP 32 conductivity meter, and for the neutral macromolecules, the total organic carbon (TOC) content was measured by means of a Shimadzu TOC V Analyzer.

In between experimental runs, the membranes were cleaned with distilled water running at maximum circulating velocity until the pure water permeate flux was regained within 5% of the initial value.

The molecular weight cut-off, MWCO, of the hybrid CA/SiO_2_ asymmetric membranes was derived from permeation experiments to neutral organic solutes and refer to the molecular weight of the solute that is 95% retained by the membrane. It is determined from the intersection of the curve of $log\left(\frac{f}{1-f}\right)$ as a function of the molecular weight of the organic neutral solutes, MW, and the line corresponding to the rejection of f=0.95. The rejection curves and MWCO determination for all membranes studied can be found in the Supplementary Data.

The statistical uncertainty associated with the measurement of the mass permeate fluxes and *f* was determined by error propagation. In the case of $L_{P}$, the uncertainty is defined by the 95% confidence intervals (CI) determined from $n_{CI}=7$ experimental points.

### 2.4. Scanning Electron Microscopy Imaging

It is known that the asymmetric CA/SiO_2_ hybrid membranes are composed of a dense skin layer with a thin porous network on top of a sub-layer with larger pores. The dense skin layer is the active layer playing a central role in the selective permeation properties of the membrane. For membranes in the UF/NF range, the pore dimensions in the active layer fall below the SEM image resolution, and only the sub-layer pores are SEM observable. The active layer pores must then be accessed by indirect methods. SEM images were recorded for the solvent exchange post-treated membranes to verify their asymmetric nature, as these membranes show significant differences relative to the remaining membranes in this study. The images were analysed with ImageJ software. For the cross-sections’ analysis, each specimen was briefly dipped in liquid nitrogen and then fractured. The samples were then placed on Al stubs using double-sided carbon tape, and a thin Au/Pd film was sputter-coated using a Quorum Technologies (Lewes, UK) sputter coater model Q150T ES. The analysis was carried out on a desktop SEM by ThermoScientific (Loughborough, UK), model Phenom ProX G6, using an acceleration voltage of 10 kV.

It is relevant to note that even with the conducting Au/Pd film, the samples were extremely sensitive to the electron beam, making the analysis very difficult and rendering the observation at high magnifications impossible. Lowering the beam energy showed hardly any influence on the observed sample degradation.

### 2.5. Water Dynamics Probed by DNMR

Deuterium NMR is an important technique for studying molecular dynamics; it relies on the deuterium nuclear magnetic moments to access, in this case, the dynamics of the heavy water molecules contained in the hydrated membranes’ pores and channels.

Pristine and post-treated dried CA/SiO_2_ membranes were immersed in deuterated water for a period of 48 h to access the water behaviour in the membranes’ porous structures. Following, the samples were prepared by enclosing a roll of D_2_O hydrated membrane in a sealed 5 mm outer diameter NMR tube. The absorption spectra, spin–lattice relaxation time, $T_{1}$ and spin–spin relaxation time, $T_{2}$, were recorded at a room temperature of 22.0±0.5 °C for all samples studied with a Bruker Avance II 300 MHz NMR spectrometer, working at a resonance frequency of 46.07 MHz for deuterium. $T_{1}$ was determined using the inversion recovery pulse sequence, and $T_{2}$ was determined with the Carr–Purcell–Meiboom–Gill (CPMG) pulse sequence [27].

The statistical uncertainty associated with the measurement of $T_{1}$ and $T_{2}$ was determined by error propagation.

## 3. Deuterium NMR Analysis

As discussed in the Introduction, the dynamics of water molecules in porous systems are affected by confinement, and these dynamics can be probed by the spin–lattice relaxation time, $T_{1}$, and the spin–spin relaxation time, $T_{2}$, measured in this study. These two NMR observables correspond, respectively, to the decay times of the longitudinal and transversal components of the nuclear magnetisation $\vec{M}$ relative to the static ${\vec{B}}_{0}$ field of the NMR spectrometer, as shown by the Bloch equations:

(2) $$\frac{d\vec{M}}{dt}=\gamma \left(\vec{M}\times \vec{B}\right)-\frac{\left(M_{x}{\vec{e}}_{x}+M_{y}{\vec{e}}_{y}\right)}{T_{2}}-\frac{\left(M_{z}-M_{0}\right){\vec{e}}_{z}}{T_{1}}$$

where $\vec{M}$ is the nuclear magnetisation of the observed element, γ is the gyromagnetic ratio of the respective nucleus, and $\vec{B}$ is the total applied magnetic field given by the sum of the static field $B_{0}{\vec{e}}_{z}$ and the radio frequency field $B_{1x}{\vec{e}}_{x}+B_{1y}{\vec{e}}_{y}$. Measurements of $T_{1}$ and $T_{2}$ can thus be used to monitor the degree of water dynamics change in a system relative to a reference system. In our case, the reference system was taken as the bulk deuterated water at room temperature used throughout the NMR study. A deeper analysis of the relaxation data was also attempted through direct modelling of the water dynamics in the hydrated membranes’ water channels and cavities, allowing the estimation of the channels and cavities’ average radius and the water molecules’ rotation correlation time at the channels’ and cavities’ surfaces. Although the model uses several assumptions that are not independently confirmed in this study, those assumptions are the same for all membranes analysed, and the model results can then be used in comparative terms between the different membranes.

Deuterium NMR relaxometry of heavy water in the membrane cavities is expected to show a strong dependence on water self-diffusion and surface interaction due to confinement, and this, allied to the fact that single exponential decays are observed for both the longitudinal and the transverse magnetisations in the systems here studied, indicates that fast exchange is occurring. As such, several models [28,29] predict relaxivities proportional to the sum of the bulk and surface relaxivities weighted by the volume fraction of the bulk versus surface-bound water:

(3) $$T_{i}^{-1}=T_{i,\;Bulk}^{-1}\left(1-\frac{Sl}{V}\right)+T_{i,\;Surface}^{-1}\frac{Sl}{V},\;with\;i=1,2$$

where *V* is the volume of the cavity, *S* is the cavity surface and *l* is the surface-bound water layer thickness. For cylindrical cavities, here assumed to represent the membrane’s water channels and with a symmetry axis normal to the membrane surface, S/V=2/R where *R* is the cylinder radius. A quantitative determination of the cylinder radius requires the determination $T_{i}$, $T_{i,\;Bulk}$ and $T_{i,\;Surface}$. In our case, we have measured $T_{i}$ and $T_{i,\;Bulk}$ (with i=1,2) for the different membranes studied and used a theoretical estimation for $T_{i,\;Surface}$.

**Figure 2:**
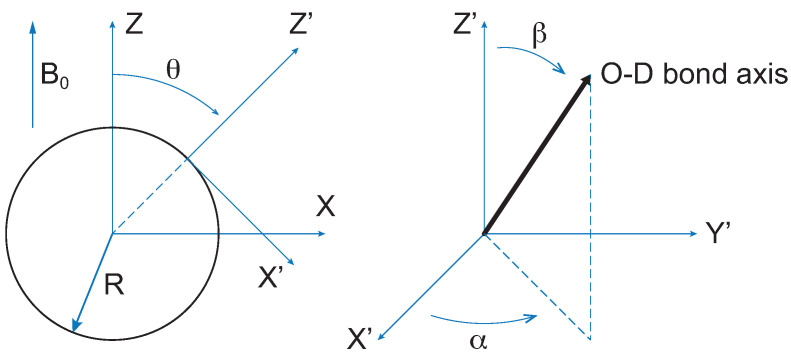
Definition of the angles θ, α and β appearing in Equation (7). (X, Y, Z) refers to the laboratory frame. (X’, Y’, Z’) is the frame associated with the water molecule diffusing in the cylindrical cavity surface. *R* is the cylinder radius. Due to the sample preparation method, the static magnetic field $B_{0}$ of the spectrometer is expected to be, on average, orthogonal to the cylinders’ axis.

NMR theory shows that the relaxivities $T_{1}^{-1}$ and $T_{2}^{-1}$ are functions of the spectral densities $J_{0}$, $J_{1}$ and $J_{2}$ [30] as:

(4) $$T_{1}^{-1}=K_{Q}\left[J_{1}\left(\omega_{0}\right)+4J_{2}\left(2\omega_{0}\right)\right]$$

(5) $$T_{2}^{-1}=K_{Q}\left[\frac{3}{2}J_{0}\left(0\right)+\frac{5}{2}J_{1}\left(\omega_{0}\right)+J_{2}\left(2\omega_{0}\right)\right]$$

where $\omega_{0}$ is the Larmor frequency, $K_{Q}=\left(\frac{3\pi^{2}}{2}\right){\left(\frac{e^{2}qQ}{h}\right)}^{2}$, and the spectral densities $J_{i}$ are Fourier transforms of correlation functions of Wigner rotation matrices $D_{lm}^{2}$ describing the orientation of the D-O bonds in the deuterated water molecules relative to a laboratory frame [30]. For DO bonds in water, $K_{Q}$ takes the value $\left(\frac{3\pi^{2}}{2}\right){\left(257\times 10^{3}\right)}^{2}\;s^{-2}$[31]. The extra contribution of $J_{0}\left(0\right)$ to $T_{2}^{-1}$ makes $T_{2}$, in general, smaller than $T_{1}$ and renders it especially sensitive to the low-frequency end of the molecular motions’ spectrum as the contribution of the spectral densities is the highest when the particular molecular motions’ characteristic frequency matches the frequency argument of the spectral densities [32]. The theoretical estimation of the spectral densities $J_{0}$, $J_{1}$ and $J_{2}$, defining the relaxivities in the cylindrical cavities’ surface, was accomplished using a model that considers the motion of the water on the surface of the cavities as dominated by two main statistically independent processes, a translational diffusion motion along the cavity surface and a local isotropic rotational diffusion motion. Following reference [32], the spectral densities take the form:

(6) $$J_{k}\left(\omega \right)=\int_{-\infty }^{\infty }\frac{\bar{V_{2,k}\left(t-\tau \right)V_{2,k}^{*}\left(t\right)}}{V_{2,0,DO}^{2}}e^{-ik\omega \tau }\;d\tau$$

where $V_{2,0,DO}=\frac{1}{2}V_{zz,DO}$ is the zero component of the gradient field irreducible spherical tensor at the deuterium site and, as indicated, is given by half of the zz component of the gradient field tensor in its principal frame at the deuterium site associated with the DO bond. The quantity $\bar{V_{2,k}\left(t-\tau \right)V_{2,k}^{*}\left(t\right)}$ is the correlation function of the k component of the gradient field’s irreducible spherical tensor associated with the DO bond in the laboratory frame. The *k* component of the gradient field’s irreducible spherical tensor is in accord with the proposed model given by:

(7) $$V_{2,k}\left(t\right)=\sum_{i=-2}^{2}D_{2,i}^{\left(2\right)}\left(0,\theta (t),0\right)D_{i,0}^{\left(2\right)}\left(\alpha (t),\beta (t),0\right)V_{2,0,DO}$$

where $D_{i,j}^{\left(2\right)}\left(\alpha ,\beta ,\delta \right)$ are the Wigner rotation matrices [33]. The angles θ(t),α(t),β(t) considered in the model are shown in Figure 2.

The correlation functions $\bar{X}\left(\tau \right)\equiv \bar{V_{2,k}\left(t-\tau \right)V_{2,k}^{*}\left(t\right)}$ were evaluated as follows [34]:

(8) $$\bar{X}\left(\tau \right)=\int X\;p_{1}\left(\theta_{0};\theta_{\tau },\tau \right)\;p_{2}\left(\alpha_{0},\beta_{0};\alpha_{\tau },\beta_{\tau },\tau \right)d\theta_{0}d\theta_{\tau }d\alpha_{0}\;sin\left(\beta_{0}\right)d\beta_{0}d\alpha_{\tau }\;sin\left(\beta_{\tau }\right)d\beta_{\tau }$$

where $p_{1}\left(\theta_{0};\theta_{\tau },\tau \right)$ is the probability that θ is $\theta_{0}$ at t=0 and $\theta_{\tau }$ at t=τ; and $p_{2}\left(\alpha_{0},\beta_{0};\alpha_{\tau },\beta_{\tau },\tau \right)$ is the probability that angles α, β take the values $\alpha_{0}$, $\beta_{0}$ at t=0 and $\alpha_{\tau }$, $\beta_{\tau }$ at t=τ. These probabilities are considered in this model to obey diffusion equations describing, respectively, the molecular translational motion on the cylindrical surface of the cavities and a spherical diffusional rotational motion of the molecule around itself, leading to:

(9) $$p_{1}\left(\theta_{0};\theta_{\tau },\tau \right)=\frac{1}{2\pi^{2}}\left[\frac{1}{2}+\sum_{m=1}^{\infty }e^{-m^{2}\frac{D_{s}}{R^{2}}\tau }cos\left[m(\theta_{\tau }-\theta_{0})\right]\right]$$

where $D_{s}$ is the molecular translational self-diffusion constant on the cavity surface and *R* is the cylinder radius identified as the average radial length of the water channels; and,

(10) $$p_{2}\left(\alpha_{0},\beta_{0};\alpha_{\tau },\beta_{\tau },\tau \right)=\frac{1}{4\pi }\sum_{l=0,\;m=-l}^{\infty ,\;l}Y_{l}^{m*}\left(\beta_{0},\;\alpha_{0}\right)Y_{l}^{m}\left(\beta_{\tau },\;\alpha_{\tau }\right)e^{-\tau \frac{l(l+1)}{6\tau_{c}}}$$

where $Y_{l}^{m}$ are the spherical harmonics and $\tau_{c}$ is the correlation time for the molecular rotation around itself [34].

The final expressions for the spectral densities, $J_{k}\left(\omega \right)$, are given by:

(11) $$J_{0}\left(0\right)=\frac{\pi }{5}\frac{1+\frac{D_{s}\tau_{c}}{R^{2}}}{4\frac{D_{s}}{R^{2}}+\tau_{c}^{-1}}$$

(12) $$J_{1}\left(\omega \right)=\frac{\pi }{10}\left[\frac{\frac{D_{s}}{R^{2}}+\tau_{c}^{-1}}{{\left(\frac{D_{s}}{R^{2}}+\tau_{c}^{-1}\right)}^{2}+\omega^{2}}+\frac{\frac{4D_{s}}{R^{2}}+\tau_{c}^{-1}}{{\left(\frac{4D_{s}}{R^{2}}+\tau_{c}^{-1}\right)}^{2}+\omega^{2}}\right]$$

(13) $$J_{2}\left(2\omega \right)=\frac{2\pi }{80}\left[\frac{\frac{4D_{s}}{R^{2}}+\tau_{c}^{-1}}{{\left(\frac{4D_{s}}{R^{2}}+\tau_{c}^{-1}\right)}^{2}+{\left(2\omega \right)}^{2}}+3\frac{\tau_{c}^{-1}}{\tau_{c}^{-2}+{\left(2\omega \right)}^{2}}+4\frac{\frac{D_{s}}{R^{2}}+\tau_{c}^{-1}}{{\left(\frac{D_{s}}{R^{2}}+\tau_{c}^{-1}\right)}^{2}+{\left(2\omega \right)}^{2}}\right]$$

Inserting the spectral densities given by Equations (11)–(13) into Equations (4) and (5) and substituting those cavity surface relaxivities into Equation (3), it is possible to set a pair of equations that relate the bulk and sample measurements of water relaxation times $T_{1}$ and $T_{2}$ and the model parameters *R*, $\tau_{c}$ and $D_{s}$, which are given by:

(14) $$T_{1}^{-1}=T_{1,Bulk}^{-1}\left(1-\frac{2l}{R}\right)+\frac{2l}{R}K_{Q}\left[J_{1}\left(\omega_{0}\right)+4J_{2}\left(2\omega_{0}\right)\right]$$

(15) $$T_{2}^{-1}=T_{2,Bulk}^{-1}\left(1-\frac{2l}{R}\right)+\frac{2l}{R}K_{Q}\left[\frac{3}{2}J_{0}\left(0\right)+\frac{5}{2}J_{1}\left(\omega_{0}\right)+J_{2}\left(2\omega_{0}\right)\right]$$

where $J_{0}$, $J_{1}$ and $J_{2}$ are given, respectively, by Equations (11)–(13). Solving the set of Equations (14) and (15), one obtains an estimate for *R*, the average radial length of the water channels and $\tau_{c}$ is the molecular rotation correlation time, provided that one uses an estimate for $D_{s}$. In our case, $D_{s}$ is not known independently and was set equal to the free deuterated water self-diffusion constant at room temperature ($D_{s}=1.872$ e${}^{-9}$ m${}^{2}$s${}^{-1}$) [35]. This value attribution to $D_{s}$ is not critical because the *R* values determined are seen to be weakly dependent upon $D_{s}$, experiencing a less than 5% change for a 10-fold decrease in the imposed $D_{s}$.

The statistical uncertainty associated with $\tau_{c}$ and *R* was determined by error propagation.

## 4. Results and Discussion

### 4.1. Membranes and Selective Permeation Properties

**Figure 3:**
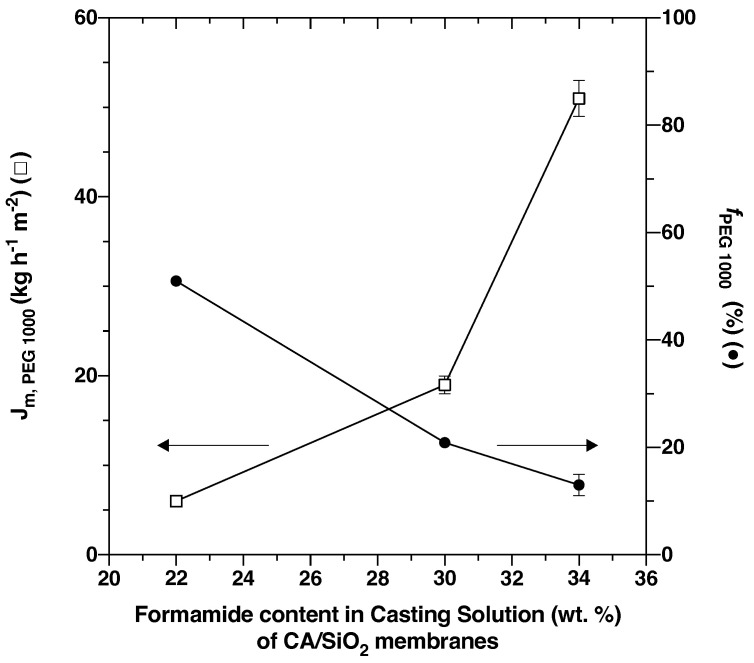
Selective permeation performance of CA/SiO_2_ membranes obtained from casting solutions with three different acetone/formamide ratios.

Figure 3 shows that for the three pristine membranes, CA/SiO_2_-22, CA/SiO_2_-30 and CA/SiO_2_-34, the apparent rejection coefficients to the solute PEG 1000, $f_{PEG\;1000}$, and the permeation fluxes of the PEG 1000 aqueous solutions, $J_{m,\;PEG\;1000}$.

Analogously to what has been evidenced for conventional wet-phase inversion CA membranes [36,37], formamide acts as an enhancing pore-forming agent and the increasing formamide content in the casting solution of 22, 30 and 34 wt.% corresponds to membrane permeate mass fluxes of 6.0±0.3, 19±1 and 51± kg h${}^{-1}$ m${}^{-2}$, and apparent rejection coefficients to PEG 1000 of 51±1, 20.9±0.3 and 13±1%, respectively.

**Figure 4:**
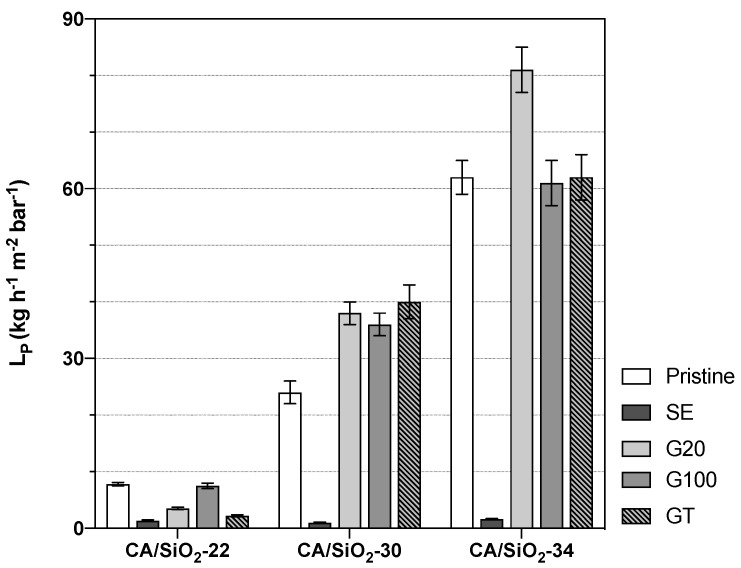
Membranes’ hydraulic permeabilities, $L_{P}$.

**Figure 5:**
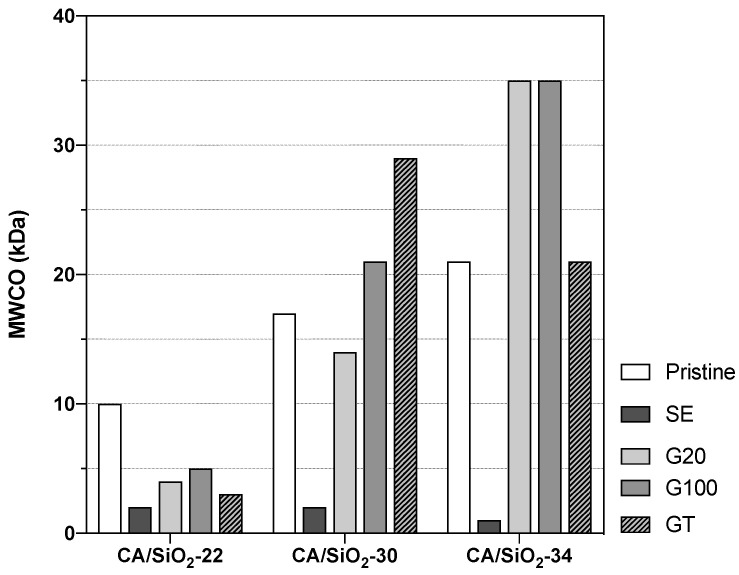
Membranes’ molecular weight cut-off, MWCO.

These three pristine membranes were subjected to drying post-treatments of solvent exchange and conditioning with surfactant solutions. The alteration of their porous structures is reflected in a wide range of hydraulic permeabilities and MWCOs, as shown in Figure 4 and Figure 5, respectively.

Owing to their distinct UF porous structures, the pristine membranes CA/SiO_2_-22, CA/SiO_2_-30 and CA/SiO_2_-34 display hydraulic permeabilities of 7.8±0.3, 24±2 and 62±3 kg h${}^{-1}$ m${}^{-2}$ bar${}^{-1}$, respectively. Their MWCOs are 10, 17 and 21 kDa, respectively.

The membranes subjected to a solvent exchange post-treatment show a drastic decrease in the hydraulic permeabilities and of their corresponding MWCOs, and this may be due to a strong pore shrinkage. On the other end, the post-treatments of conditioning the membranes with surfactant solutions yielded less-pronounced alterations of their hydraulic permeabilities and MWCOs. However, the effect of the G20, G100 and GT post-treatments resulted differently in the less permeable membranes, CA22/SiO_2_-22, than in the more permeable ones, CA/SiO_2_-30 and CA/SiO_2_-34. In fact, in the last ones, there is a general increase in $L_{P}$s and MWCOs. In contrast with that, the CA/SiO_2_-22-G20, CA/SiO_2_-22-G100 and CA/SiO_2_-22-GT membranes have lower values of $L_{P}$s and MWCOs when compared to the values of the corresponding pristine membranes.

**Figure 6:**
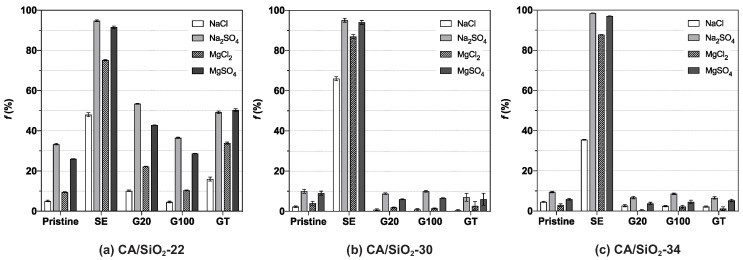
Apparent rejection coefficient to salts, *f*, of (**a**) CA/SiO_2_-22, (**b**) CA/SiO_2_-30 and (**c**) CA/SiO_2_-34 membranes.

Figure 6 displays the apparent rejection coefficients to salts, NaCl, MgCl_2_, Na_2_SO_4_ and MgSO_4_.

The UF membranes are known to exhibit a negligible degree of retention to salts. This behaviour is promptly observed in the two most permeable pristine membranes, CA/SiO_2_-30 and CA/SiO_2_-34, which evidence apparent rejection coefficients to all salts below 11%. Contrastingly, the least-permeable pristine membrane, CA/SiO_2_-22, reveals a different selective permeation pattern characterised by comparably slightly higher rejection coefficients to Na_2_SO_4_ and MgSO_4_, being equal to 33.4±0.4% and 26.02±0.15%, respectively. These findings suggest that, although its MWCO is comparably smaller than those of the pristine CA/SiO_2_-30 and CA/SiO_2_-34 membranes, their higher negative surface charge may play an important role in determining the higher rejection of the bivalent sulphate ions.

Additionally, the membranes’ permselectivity to salts after the post-treatment by SE is characterised by increased values of the apparent rejection coefficients to the solutes. Of these, the rejection is almost total to salts containing the sulphate ($SO_{4}^{2-}$) ion and higher than 34% for those containing the chloride ($Cl^{-}$) ion. These results may be attributed to the modification of the porous structure of the membranes, verifiable by their MWCO reduction, that occurred after the treatment. Consequently, the observed degree of retention on SE-treated membranes may be explained by both a combination of size exclusion mechanisms, as their MWCO is very small, and interactions established between the membrane’s charged surface and the charged solutes. These permeation profiles are very often associated with the selective permeation characteristics of nanofiltration [38], which are denoted mainly by the capability to differentiate between ionic species. On the other hand, the conditioning treatment with surfactant solutions (G20, G100 and GT) had minimal effects on the membranes’ selective permeation to salts.

### 4.2. SEM Imaging

**Figure 7:**
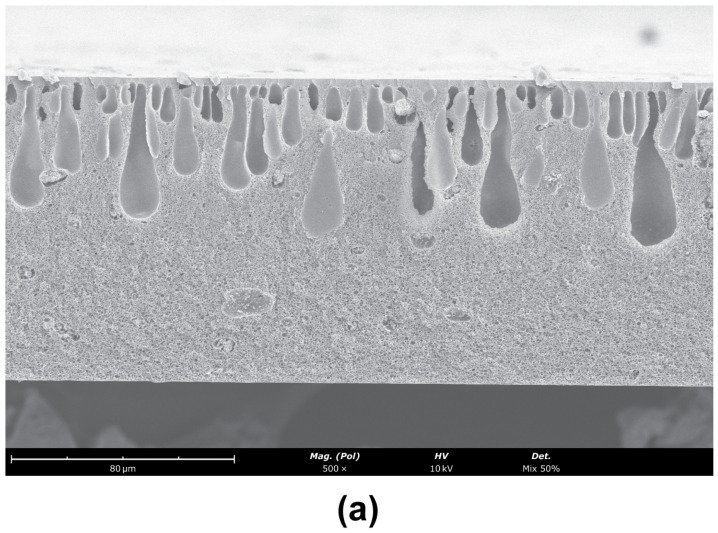

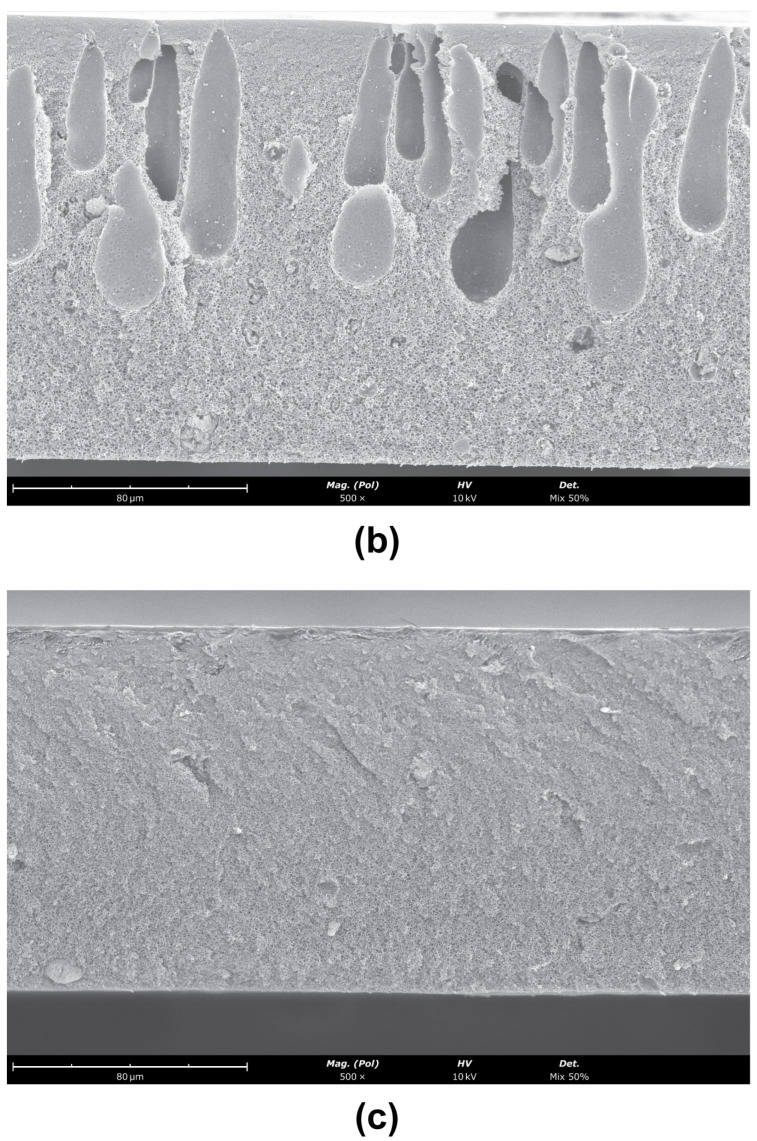
SEM images of the cross-sections (magnification ×500) of (**a**) CA/SiO_2_-22-SE, (**b**) CA/SiO_2_-30-SE and (**c**) CA/SiO_2_-30-SE. The active layer is located at the top surface of the membrane.

The SEM images obtained for the CA/SiO_2_-22-SE, CA/SiO_2_-30-SE and CA/SiO_2_-34-SE membranes are shown in Figure 7.

**Figure 8:**
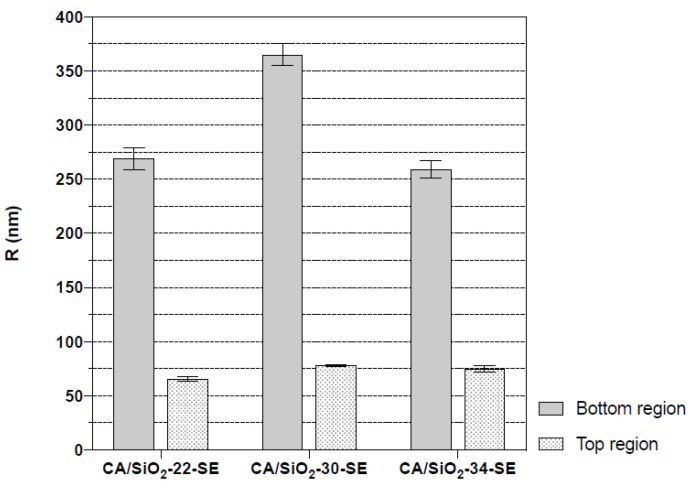
Average cavity radius of CA/SiO_2_-SE membranes in a region near the membrane’s bottom and close to the membrane’s top active layer.

The asymmetric nature of these membranes is identified in the cross-section of the membranes, with a significant decrease in pore dimensions towards the active layer. The active layer pores are not observable. Finger-like macro-void structures detected in the CA/SiO_2_-22-SE and CA/SiO_2_-SE membranes seem to have no significant impact on the measurements carried out. Figure 8 reports the average cavity radius extracted from the SEM images near the bottom and close to the active layer at the top of the membrane, obtained with ImageJ software. The data show the decreasing trend of the pore radius towards the active membrane layer in all membranes.

### 4.3. Water Dynamics in Hydrated Membranes as Seen by DNMR

**Figure 9:**
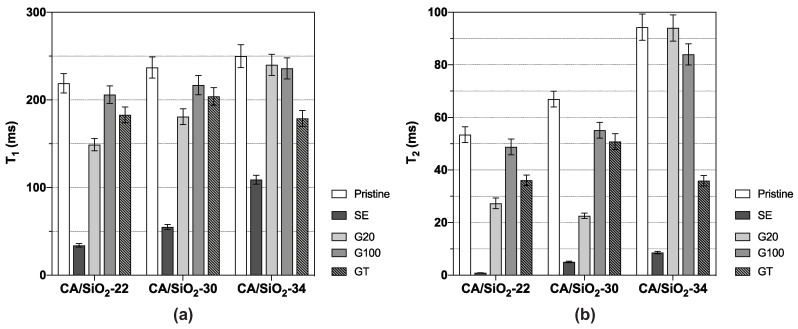
(**a**) Spin–lattice and (**b**) spin–spin relaxation times, $T_{1}$ and $T_{2}$, respectively, of CA/SiO_2_ membranes.

Two distinct spectral components compatible with two distinct water populations were identified in membranes CA/SiO_2_-30 and CA/SiO_2_-34; this fully complies with the asymmetric structure of these membranes, which includes a dense skin layer with a thin-porous network on top of a sublayer with larger pores. The results obtained for $T_{1}$ and $T_{2}$ from the spectral component more affected by the hybrid membrane matrix are shown in Figure 9. The reported $T_{1}$ and $T_{2}$ values are well below those measured for pure deuterated water at the same temperature, being $T_{1}=T_{2}=410\pm 20$ ms, respectively, indicating that the interaction between the water molecules and the membrane’s polymer matrix significantly affects the water dynamics.

Due to the asymmetric nature of the CA/SiO_2_ hybrid membranes, a significant spread in sizes of the water-filled membrane cavities and channels is expected across the membranes’ cross-section. As such, the DNMR observables are expected to convey average values over the sizes’ distribution.

**Figure 10:**
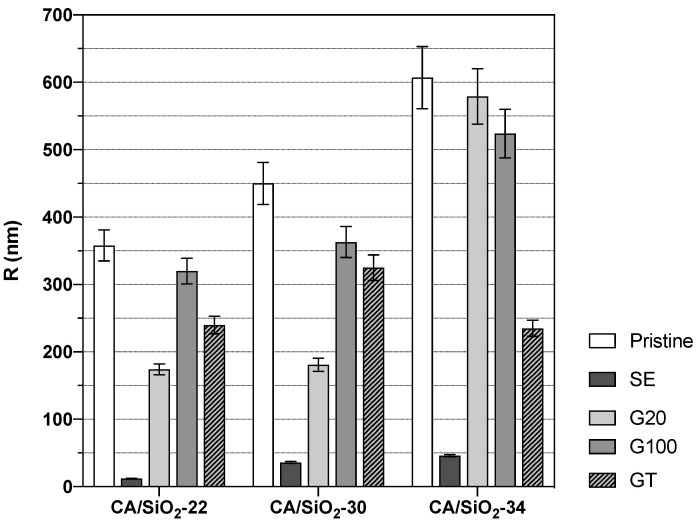
Average radial length of the water channels determined from the DNMR relaxation times $T_{1}$ and $T_{2}$.

The $T_{1}$ and $T_{2}$ values reported in Figure 9 can be compared in relative terms with the results from the hydraulic permeability and molecular weight cut-off obtained from the same membranes. The solvent exchange-treated membranes show the largest change in water dynamics relative to the reference (bulk water) amongst all membranes. This fact could arise from more effective confinement of the water molecules in the SE membranes. Hydraulic permeability and molecular weight cut-off in those membranes take the lowest values amongst all membranes studied, thus indicating a strong pore shrinkage, and this fact perfectly matches the higher degree of confinement detected by DNMR relaxation. Each of the three pristine and G20 and G100 post-treated membranes exhibit similar $T_{1}$s and $T_{2}$s, with G20 showing some dispersion in $T_{2}$. Comparing these results with those from hydraulic permeability and molecular weight cut-off, one can see that for each pristine membrane, CA/SiO_2_-22, CA/SiO_2_-30 and CA/SiO_2_-34, the corresponding ones post-treated by G20 and G100 exhibit similar hydraulic permeabilities, whilst the disperse molecular weight cut-off values are higher. This similarity between the pristine and the corresponding ones post-treated by G20 and G100 is confirmed for the $T_{1}$ values and also $T_{2}$ values, albeit to a lower degree. Another very clear tendency shared by the hydraulic permeability and molecular weight cut-off measurements with the $T_{1}$ and $T_{2}$ measurements is their value increase with the increase in the pore dimensions or decrease in the confinement effect, and this is verified for all post-treatments. This common tendency originates from the fact that the increase in the membrane pore dimensions leading to higher hydraulic permeability and molecular weight cut-off is associated with weaker confinement effects over the water dynamics and, therefore, generating larger $T_{1}$ and $T_{2}$ values. Using the $T_{1}$ and $T_{2}$ values reported in Figure 9 and the NMR model presented earlier, it was possible to determine values for *R* and $\tau_{c}$ for each membrane. The values found for *R* are reported in Figure 10, while the $\tau_{c}$ values appear in Table 2.

For the pristine membranes, the $\tau_{c}$ decreases from the less permeable to the more permeable membranes. The membranes subjected to a solvent exchange post-treatment display high values of $\tau_{c}$ and are particularly pronounced for the less permeable membrane, CA/SiO_2_-22-SE. These results point to a slowing down of the water rotational diffusion on the channel’s surface correlated with tighter confinement.

This NMR analysis enables the determination of an average radial length of the water channels associated with the most restricted water component, but due to the asymmetric nature of the membranes, these average values may be quite different from the pore radius at the membranes’ active layer surface. Despite this, it is expected that these measurements carry significant information on the membranes’ water channels’ radial length and might thus be useful in comparing different membranes as selective permeation agents.

From Figure 10, it is possible to see a general increase in the average radial length of the water channels as one moves from membranes CA/SiO_2_-22 to CA/SiO_2_-30 and to CA/SiO_2_-34 and the corresponding ones after SE, G20, G100 and GT post-treatments. These results go along with the permeation data obtained that evidenced an increase, progressively, in the convective permeation properties.

Further, the consequence of the drying process can be followed as one moves from the pristine membranes to the glycerol post-treated membranes (G100, GT, G20) and, finally, the SE treated membranes. The G100 treated membranes do not display significant alterations relative to the pristine membranes. Additionally, the GT and the G20-treated membranes show some alteration, relative to the pristine CA/SiO_2_ membranes in the direction of water channel radial length shrinkage, but does not translate into hydraulic permeability reduction. The SE-treated membranes exhibit a very significant decrease in average water channel radial length compatible with a highly reduced hydraulic permeability. Measurements of pore radius in SE post-treated membranes, obtained from SEM image analysis, were reported in Figure 8, although those measurements do not include the pores in the membrane active layer as discussed previously. On the contrary, the DNMR-obtained average pore radius is dominated by the pores in the active layer since those pores experience the highest degree of confinement for the water molecules. As such, the average pore dimensions obtained by NMR are significantly lower than those registered with SEM imaging. This study gives a consistent indication that deuterium NMR relaxation times are reliable tools to monitor the effects of the post-treatments on the porous structure of the hybrid CA/SiO_2_ asymmetric membranes.

## 5. Conclusions

In this work, the synthesis and characterisation of three UF CA/SiO_2_ monophasic membranes with very distinct asymmetric porous structures allowed the evaluation of the effect of membrane post-formation treatments, by solvent exchange and conditioning in surfactant mixtures, on their structure modification and permeation properties. The assessment of permeation characteristics was carried out in terms of hydraulic permeability, apparent rejection coefficients to salts and neutral organic macromolecules, as well as the determination of MWCO of the membranes, conjointly with the study of water dynamics probed by DNMR within the membranes’ porous structures.

Based on the characterisation of the permeation properties, it can be concluded that the solvent exchange treatment alters the membranes’ porous structures by shrinking their pores. This evidence is associated with a decrease in the pure water permeate fluxes, a total rejection of bivalent salts and lower MWCO, as well as a significant decrease in relaxation times, $T_{1}$ and $T_{2}$, and, consequently, in radial length of the membrane water channels, when compared to pristine membranes. Consequently, the permeation pattern of SE-treated membranes is analogous to those of NF membranes.

On the other side, the conditioning with surfactant mixtures presented minimal dissimilarities in the membranes’ permeation properties. More specifically, the post-treated membranes with pure glycerol solutions, G100, are characterised by a permeation performance that is very alike to those of pristine membranes. This may be attributed to their resemblances in the porous structures, as shown by the observed relaxation rates and average water channel radial lengths in both treated and pristine membranes.

## Figures and Tables

**Table 1:** Film casting solution compositions and casting conditions of CA/SiO_2_ hybrid membranes.

Casting Solution Composition (wt. %)			
**Membrane**	**CA/SiO_2_-22**	**CA/SiO_2_-30**	**CA/SiO_2_-34**
CA	16.4	16.4	16.4
Formamide	21.3	29	32.9
Acetone	58.8	51.1	47.2
TEOS (SiO_2_ precursor)	3	3	3
H_2_O	0.5	0.5	0.5
HNO_3_	4 drops (pH ≈ 2)	4 drops (pH ≈ 2)	4 drops (pH ≈ 2)
**Casting Conditions**			
Temperature of casting solution (°C)		20–25	
Temperature of casting atmosphere (°C)		20–25	
Relative humidity of casting atmosphere (%)		40–50	
Solvent evaporation time (min)		0.5	
Gelation medium		Ice-cold deionised water (2 h)	

**Table 2:** Correlation times $\tau_{c}$ (ns) for the molecular rotational diffusion motion.

Membranes	CA/SiO_2_-22	CA/SiO_2_-30	CA/SiO_2_-34
Post-Treatments			
Pristine	9.7±0.8	9.2±0.9	7.5±0.9
SE	61±4	14±0.6	8.4±0.5
G20	10±0.6	14±0.9	7±0.8
G100	9.6±0.8	9.4±0.8	7.5±0.8
GT	10±0.7	9.2±0.8	10±0.7

## Data Availability

The original data can be found at: https://fenix.tecnico.ulisboa.pt/homepage/ist167997, accessed on 25 April 2022.

## Supplementary Materials

The following are available online at https://www.mdpi.com/article/10.3390/membranes12060559/s1. Figure S1: Variation of the apparent rejection coefficient, f, with neutral organic solute molecular weight and determination of the MWCO for the CA/SiO2-22 membranes. Figure S2. Variation of the apparent rejection coefficient, f, with neutral organic solute molecular weight and determination of the MWCO for the CA/SiO2-30 membranes. Figure S3. Variation of the apparent rejection coefficient, f, with neutral organic solute molecular weight and determination of the MWCO for the CA/SiO2-34 membranes.

## Abbreviations

The following abbreviations are used in this manuscript:

CA	Cellulose acetate
RO	Reverse osmosis
NF	Nanofiltration
UF	Ultrafiltration
CA/SiO_2_	Cellulose acetate/silica
NMR	Nuclear magnetic resonance
D	Deuterium
D_2_O	Deuterated water
DNMR	Deuterium nuclear magnetic resonance
SEM	Scanning electron microscopy
SE	Solvent exchange
G20	Surfactant conditioning with an aq. sol. of glycerol 20 vol. %
G100	Surfactant conditioning with pure glycerol
GT	Surfactant conditioning with an aq. sol. of glycerol 20 vol. % and triton x-100 4 vol. %
