# Supplementary material for: Tailoring the Selective Permeation Properties of Asymmetric Cellulose Acetate/Silica Hybrid Membranes and Characterisation of Water Dynamics in Hydrated Membranes by Deuterium Nuclear Magnetic Resonance
Source: Membranes (Basel). 2022 May 28;12(6):559. doi: 10.3390/membranes12060559 (PMC9229797; doi:10.3390/membranes12060559)
Supplement: Supplementary file 1 [file membranes-12-00559-s001.zip › membranes-1722418-supplementary.pdf]

membranes

Article

Tailoring the Selective Permeation Properties of Asymmetric Cellulose Acetate/Silica Hybrid Membranes and Characterisation of Water Dynamics in Hydrated Membranes by Deuterium Nuclear Magnetic Resonance

Miguel P. da Silva [1,2], Maria J. Beira [1,3], Isabel D. Nogueira [4], Pedro J. Sebastiao [1,3] and Joao L. Figueirinhas [1,3] and Maria Norberta de Pinho [1,2]*

1 Center of Physics and Engineering of Advanced Materials (CeFEMA), Laboratory for Physics of Materials and Emerging Technologies (LaPMET), Instituto Superior Tecnico (IST), Universidade de Lisboa (ULisboa), Av. Rovisco Pais 1, 1049-001 Lisboa, Portugal; miguel.pereira.da.silva@tecnico.ulisboa.pt (M.P.d.S.); maria.beira@tecnico.ulisboa.pt (M.J.B.); pedro.jose.sebastiao@tecnico.ulisboa.pt (P.J.S.);joao.figueirinhas@tecnico.ulisboa.pt (J.L.F.)
2 Department of Chemical Engineering (DEQ), Instituto Superior Tecnico (IST), Universidade de Lisboa (ULisboa), Av. Rovisco Pais 1, 1049-001 Lisboa, Portugal
3 Department of Physics (DF), Instituto Superior Tecnico (IST), Universidade de Lisboa (ULisboa), Av. Rovisco Pais 1, 1049-001 Lisboa, Portugal
4 MicroLab, Instituto Superior Tecnico (IST), Universidade de Lisboa (ULisboa), Av. Rovisco Pais 1, 1049-001 Lisboa, Portugal; isabel.nogueira@tecnico.ulisboa.pt (I.D.N.)
* Correspondence: Correspondence: marianpinho@tecnico.ulisboa.pt (M.N.d.P.); Tel.: +351-218-417-488

A Supplementary Data

Figures S1, S2 and S3 show the rejection curves of reference neutral organic solutes and the determination of the MWCO for the CA/SiO_2-22, CA/SiO_2-30 and CA/SiO_2-34 membranes, respectively

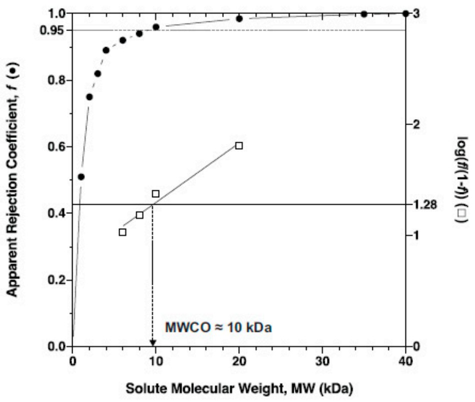

(a.1) CA/SiO_2-22 Pristine

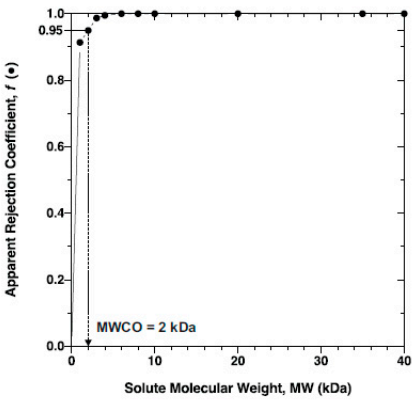

(a.2) CA/SiO_2-22-SE

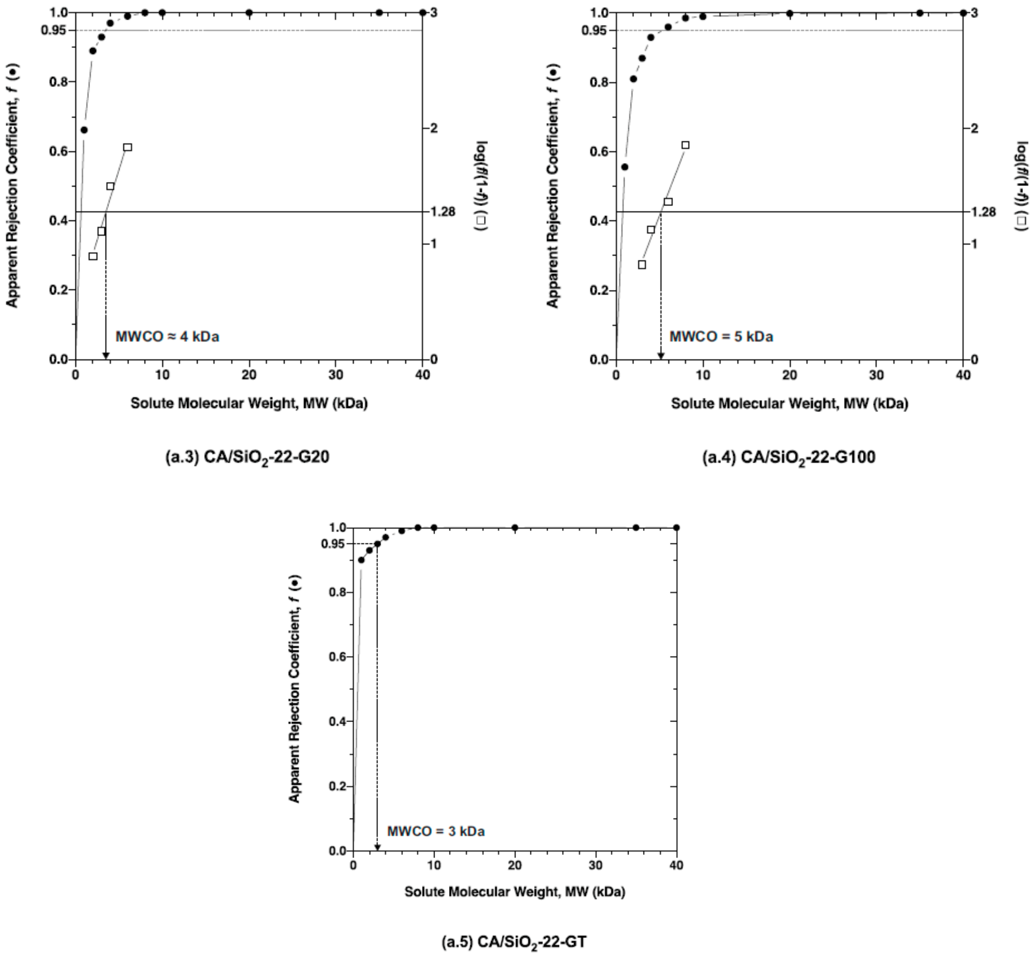

Figure S1. Variation of the apparent rejection coefficient, f, with neutral organic solute molecular weight and determination of the MWCO for the CA/SiO2-22 membranes.

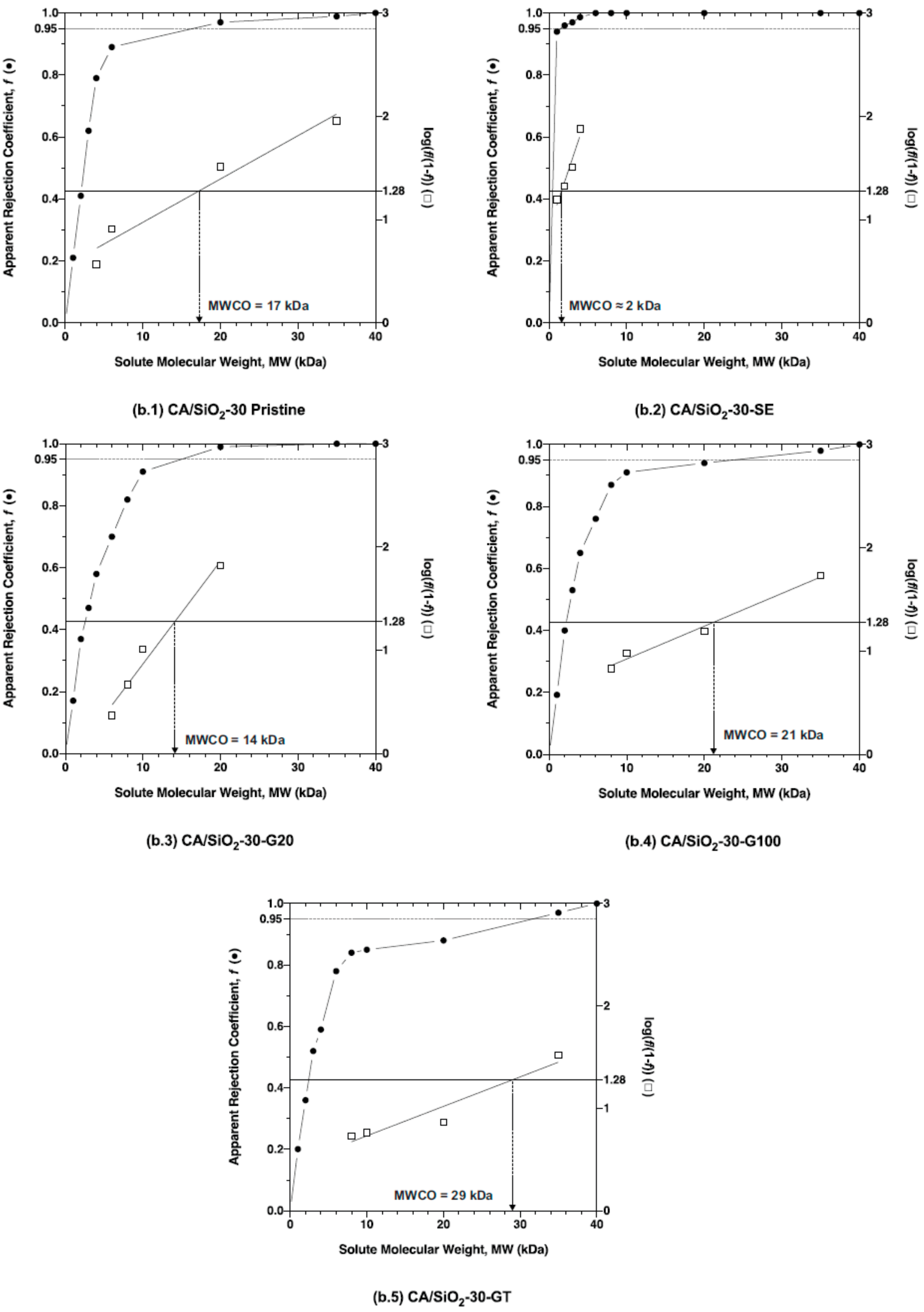

Figure S2. Variation of the apparent rejection coefficient, f, with neutral organic solute molecular weight and determination of the MWCO for the CA/SiO2-30 membranes.

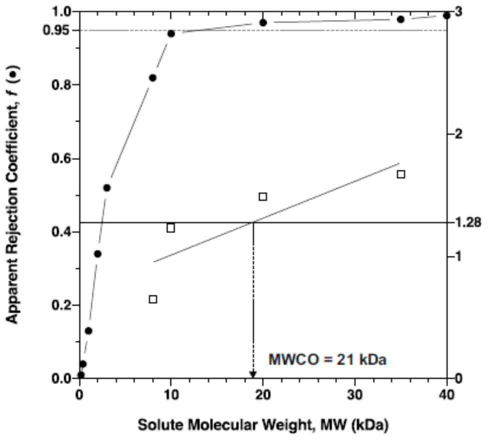

(c.1) CA/SiO_2-34 Pristine

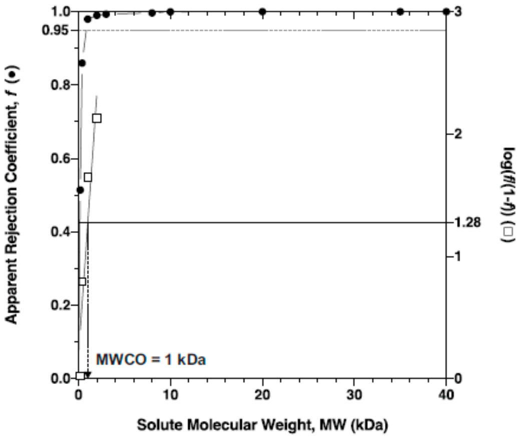

(c.2) CA/SiO_2-34-SE

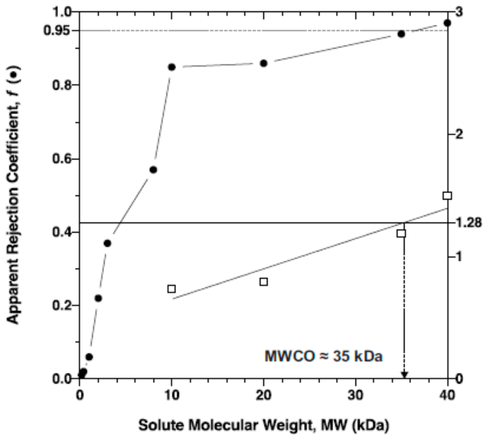

(c.3) CA/SiO_2-34-G20

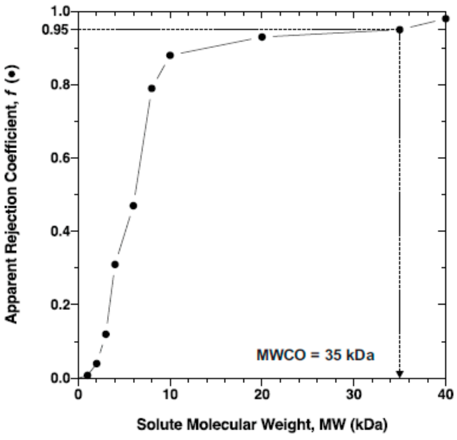

(c.4) CA/SiO_2-34-G100

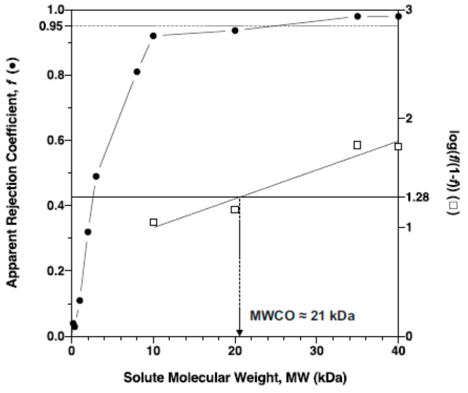

(c.5) CA/SiO_2-34-GT

Figure S3. Variation of the apparent rejection coefficient, f, with neutral organic solute molecular weight and determination of the MWCO for the CA/SiO2-34 membranes.
